# Risk versus reward: host dependent parasite mortality rates and phenotypes in the facultative generalist *Triphysaria versicolor*

**DOI:** 10.1186/s12870-019-1856-1

**Published:** 2019-08-01

**Authors:** Loren A. Honaas, Sam Jones, Nina Farrell, William Kamerow, Huiting Zhang, Kathryn Vescio, Naomi S. Altman, John I. Yoder, Claude W. dePamphilis

**Affiliations:** 1 Intercollege Graduate Program in Plant Biology, Huck Institutes of the Life Sciences, The Pennsylvania State University, University Park, PA 16802 USA; 2 Department of Biology, The Pennsylvania State University, University Park, PA 16802 USA; 3 Department of Statistics and Huck Institutes of the Life Sciences, The Pennsylvania State University, University Park, PA 16802 USA; 4 Department of Plant Sciences, University of California, Davis, CA 95616 USA; 5 Present address: Physiology and Pathology of Tree Fruits Research, USDA - Agricultural Research Service, Wenatchee, WA 98801 USA

## Abstract

**Background:**

Parasitic plants engage in a complex molecular dialog with potential host plants to identify a host and overcome host defenses to initiate development of the parasitic feeding organ, the haustorium, invade host tissues, and withdraw water and nutrients. While one of two critical signaling events in the parasitic plant life cycle (germination via stimulant chemicals) has been relatively well-studied, the signaling event that triggers haustorium formation remains elusive. Elucidation of this poorly understood molecular dialogue will shed light on plant-plant communication, parasitic plant physiology, and the evolution of parasitism in plants.

**Results:**

Here we present an experimental framework that develops easily quantifiable contrasts for the facultative generalist parasitic plant, *Triphysaria*, as it feeds across a broad range of diverse flowering plants. The contrasts, including variable parasite growth form and mortality when grown with different hosts, suggest a dynamic and host-dependent molecular dialogue between the parasite and host. Finally, by comparing transcriptome datasets from attached versus unattached parasites we gain insight into some of the physiological processes that are altered during parasitic behavior including shifts in photosynthesis-related and stress response genes.

**Conclusions:**

This work sheds light on *Triphysaria’s* parasitic life habit and is an important step towards understanding the mechanisms of haustorium initiation factor perception, a unique form of plant-plant communication.

**Electronic supplementary material:**

The online version of this article (10.1186/s12870-019-1856-1) contains supplementary material, which is available to authorized users.

## Background

*Triphysaria versicolor* is a model parasitic plant in the family Orobanchaceae [1, 2], a family that represents one of a likely 12 independent origins of parasitism in flowering plants [3, 4]. *T. versicolor* is a facultative parasite, and a generalist that can parasitize a wide range of monocot and eudicot hosts, both in nature [5], and in the laboratory [6]. Other members of this family are a primary constraint to African agriculture [7], infesting 40% of all cereal crops in sub-Saharan Africa [8], and causing an estimated $US 10 billion in crop damage annually [9, 10]. The Orobanchaceae also provide unique opportunities to study parasitism as it is the only plant family with the full range of parasitic lifestyles [11], plus a fully autotrophic sister lineage, *Lindenbergia* [12]. In addition to their usefulness for understanding the evolution of parasitism (and thus novel traits, [13, 14]), these plants display extremes of physiology and development that can help us understand many facets of plant biology. For example, strigolactones, long known as germination stimulants [15] for parasitic members of Orobanchaceae, were discovered in 2008 to be important plant hormones [16, 17], the likely receptors for which have been recently identified [18].

Strigolactones are also important signaling molecules perceived by arbuscular mycorrhizal (AM) fungi during symbiosis [19], suggesting that parasitic plants have evolved to eavesdrop on the molecular dialogue between potential hosts and symbiotic fungi [20]. Interruption of this dialogue has been identified as one of the potential control points for weedy parasitic Orobanchaceae [21–23]. However, the impact of altering strigolactone levels in the plant and in the rhizosphere as part of an effort to control parasitic weeds is still being explored. This is complicated by recent work reporting protective effects of AM fungi against *Striga hermonthica* in Sorghum [24]. Another potential point of control is post attachment physiology of the parasite [10]. Post attachment resistance traits are usually polygenic and breeding programs have targeted these modes of resistance, though only partial and short-term resistance has been achieved [10]. A third point of control is the mechanism by which parasitic plants initiate haustorium formation [1], including the perception of haustorium inducing factors (HIFs). Raw host root exudates contain active HIFs including various quinones, hydroquinones, phenolic acids and flavonoids [25]. It is likely that the considerable redundancy in host derived HIFs contributes to the broad host range of parasitic Orobanchaceae [25]. It also presents the possibility that a complex HIF profile conveys host quality information, providing a point at which the parasite can evaluate its host in preparation for attachment [25]. The mechanism of this process is largely unknown, save the following observations: 1) structurally diverse active HIFs all have a narrow window of redox potentials [25], 2) the quinone reductase TvQR1 is important for haustorium initiation in *Triphysaria* and acts very early in HIF perception [25, 26], and 3) that TvPirin is necessary for haustorium formation [27]. Interestingly, TvQR1 has a much greater allelic diversity than TvPirin, with the highest diversity in a protein domain that determines substrate specificity [28]. This diversity may help explain *Triphysaria’s* ability to respond to a wide variety of host root exudates and hence feed across a broad host range.

In obligate parasites like *Striga* the commitment to haustorium formation (i.e. haustoriogenesis) is made at germination, because even though separate signaling events must occur to initiate haustoriogenesis, seed resources are quickly exhausted and provide only a few days of resources to effect successful attachment to a host root, without which the seedling dies [29]. Therefore, it is critical to coordinate haustorium formation with radicle growth and with regard to the proximity and orientation to the potential host root via the perception of HIFs. In contrast, the commitment to haustoriogenesis in facultative hemiparasites, like *Triphysaria*, occurs via HIF perception by roots of ostensibly *free-living plants*. The facultative generalist parasite must also evaluate potential hosts during the free-living phase of growth to identify high quality versus low quality hosts. The general lack of self-haustorium formation, plus the reduced rate of haustorium formation on congeneric plants (i.e. *T. eriantha*), compared to *Arabidopsis thaliana,* shows that *Triphysaria* has the ability to evaluate host quality [30]. Because *Triphysaria* does not require a host-derived germination simulant, host evaluation is uncoupled from germination, making the facultative generalist a useful model for characterization of HIF perception processes in parasitic plants. Importantly, the host range of *Triphysaria* overlaps with that of the weedy Orobanchaceae and provides a framework for discovery of host recognition and evaluation machinery that is shared family-wide. Previous work has shown that another facultative parasitic Orobanchaceae, *Castilleja densiflora* (syn. *Orthocarpus densiflora*) displays host dependent floral phenotypes [31] as well as host dependent survivorship [32]. Furthermore, phenotypic transitions to more vigorous growth, thought to occur after successful attachment, have been noted [32, our unpublished field observations]. Therefore, we hypothesized that *Triphysaria* would display host dependent phenotypes during interactions with various hosts that we could magnify by growing the parasite on distantly related plants that span the parasite’s host range.

We selected a group of experimentally tractable host plant genera, based in part upon the survey by Thurman [5], that includes three eudicots (*Arabidopsis*, *Medicago,* and *Solanum*) and three monocots (*Zea, Oryza,* and *Juncus*). Here we describe experiments that reveal clines of easily quantifiable parasite phenotypes displayed by *Triphysaria* while it fed across its host range. These phenotypes suggest that the generalist parasite may have the ability to evaluate host quality, and our framework provides a means to evaluate parasite success nondestructively throughout the parasite’s life cycle. Surprisingly, we found that phenotypes that indicate enhanced parasite vigor were strongly correlated with low parasite survivorship. We also show that direct parasite-host contact, not just host root exudate, is necessary for the development of a distinct growth phenotype. Finally, we developed image-based analytics that recapitulate destructive measurements that will allow us to capture phenotypic transitions during host-parasite interactions with non-destructive time course measurements.

## Results

### Co-culture across *Triphysaria’s* host range

**Fig. 1:**
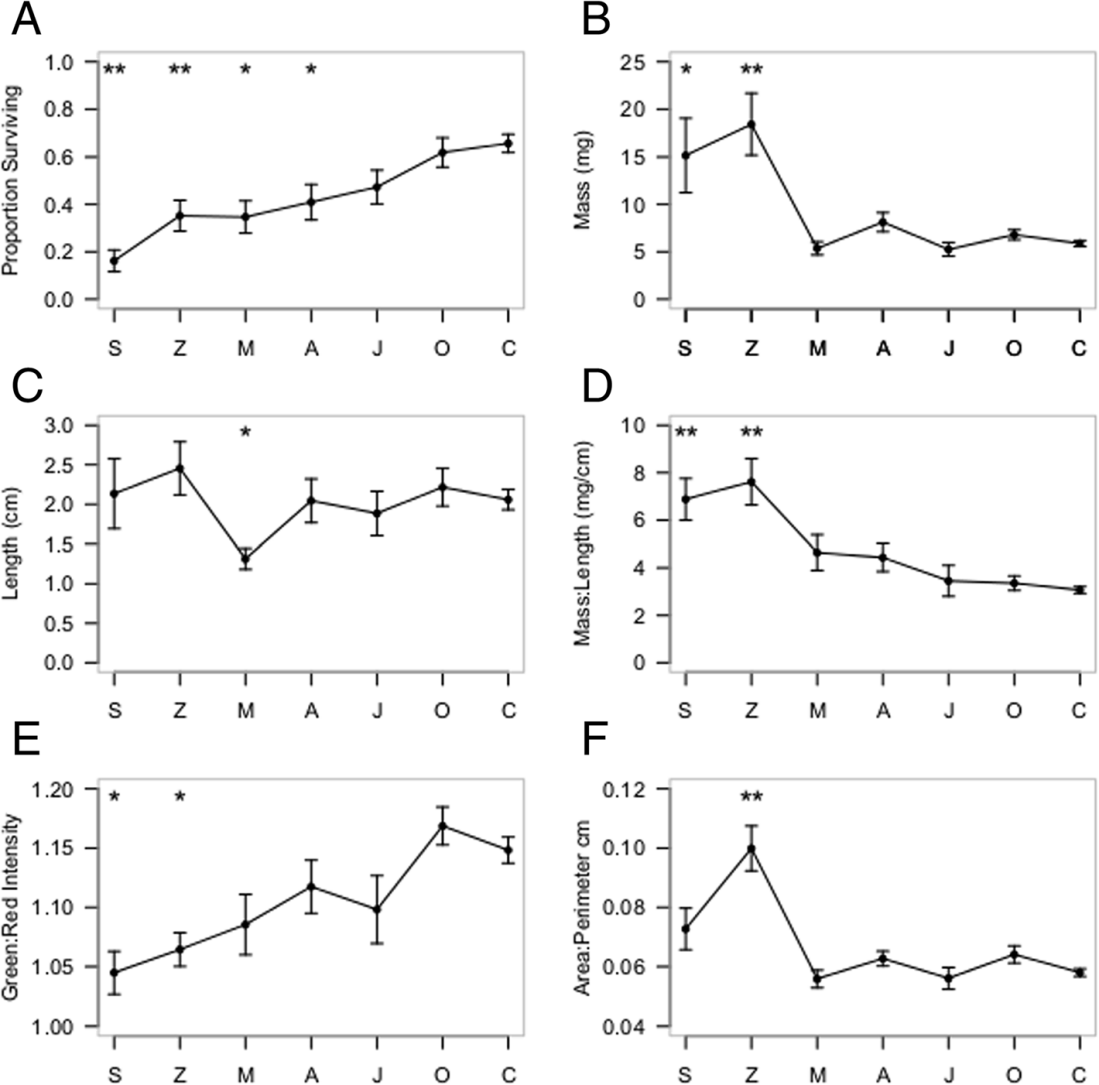
The characteristics of *Triphysaria* grown across its host range are significantly different from host-free plants and are often highly correlated. ANOVA (Dunnett-Hsu correction) statistical significance compared to the control *p < 0.05 and **p < 0.01. S=*Solanum*, Z = *Zea*, M = *Medicago*, A = *Arabidopsis*, J = *Juncus*, O=*Oryza*, C = host-free control. Pearson’s R^2^ for A vs. E = 0.86, D vs. E = 0.69

The host range co-culture experiment was monitored daily and survivorship of *Triphysaria* was recorded weekly by counting surviving individuals. By the 5th week of greenhouse co-culture, parasites in the *Solanum* (*p* > 0.001), *Zea* (*p* = 0.001), *Medicago* (*p* = 0.003), and *Arabidposis* (*p* = 0.034) pots showed significantly fewer surviving individuals than the control pots (Fig. 1a.) Because the trend appeared to be toward very low parasite survivorship in the experimental treatments, the greenhouse experiment was ended and several measurements, some destructive, were made to discover host dependent parasite phenotypes. Furthermore, because some of these patterns were very surprising, we employed very conservative statistics to avoid false positives. Simple parasite growth parameters were significantly different than the control with hosts that induced the lowest survivorship. This included higher dry mass (Fig. 1b: for *Zea p* < 0.001 & *Solanum p* = 0.026) indicating that even though the parasites were less likely to survive with *Solanum* and *Zea* hosts, survivors accrued more tissue than free-living individuals.

**Fig. 2:**
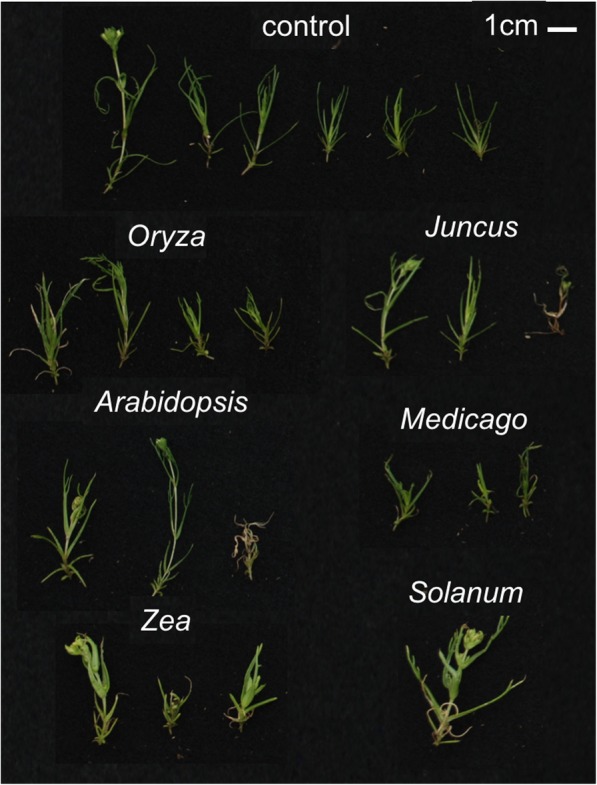
*Triphysaria* displays host dependent phenotypes**.** Representative images of *Triphysaria* showing the average number of surviving plants in each treatment, plus controls. The host genus is listed above each set of parasites. The control plants were grown in identical conditions, in an identical circular arrangement, but without hosts

Compared to the gracile control plants, hosts which induced the highest parasite mortality also induced a novel phenotype – the survivors were “pale and plump” (Fig. 2: e.g. *Solanum* and *Zea* compared to the control), apparently due to shortened internodes and altered leaf morphology. We attempted to quantify the “plump” phenotype by integrating aspects of the growth parameter data. We integrated the dry mass (Fig. 1b) and plant height measurements (Fig. 1c) to produce a ratio to quantify the “plump” phenotype (Fig. 1d). Compared to the control, there were significant differences for *Triphysaria* grown with *Zea* (*p* = < 0.001) and *Solanum* (*p* = 0.0083). We hypothesized that the paleness of the plants was due to reduced chlorophyll content, therefore we attempted to quantify the “pale” phenotype by estimating the red:green ratio of plants (as described in [33] because this method was useful to estimate changes in chlorophyll content in senescing wheat). We analyzed photographs of all surviving individuals (See Fig. 2 for representative images from each treatment). The red:green ratio was significantly different than the host-free control for *Triphysaria* grown with *Solanum* (*p* = 0.0247) and *Zea* (*p* = 0.0162) (Fig. 1e). The gradation of paleness was strongly correlated (R^2^ = 0.86) with survivorship rates (Fig. 1a) and moderately so with the mass length ratio (Fig. 1e, R^2^ = 0.69) suggesting that these phenotypes may be related. Considering all evidence together, the trend for *Triphysaria* grown with known hosts was fewer surviving individuals that were hardier and ostensibly less autotrophic.

Because analyzing the plant images allowed us to quantify the “greenness” of plants, thereby confirming visual observations and showing significant experimental contrasts, we attempted to recapitulate the “plumpness” (e.g. mass/height ratio) of plants as well. By analyzing each photograph to outline each plant, we generated perimeter to area ratios. This approach recapitulated our plant mass measurements (R^2^ = 0.87; see Fig. 1b vs. 1f) and showed a significant difference of parasites grown with *Zea* versus the host-free control. The correlation with the mass/height ratio was good, but lower (R^2^ = 0.72) indicating further refinement of the method is needed to accurately capture the experimental contrasts, namely a way to estimate plant height in a high throughput manner. Importantly, these image-based analytics showed us significant phenotypic differences between at least partly heterotrophic parasites and the autotrophic parasite controls.

### Co-culture with *Solanum* and subirrigation of *Triphysaria*

**Fig. 3:**
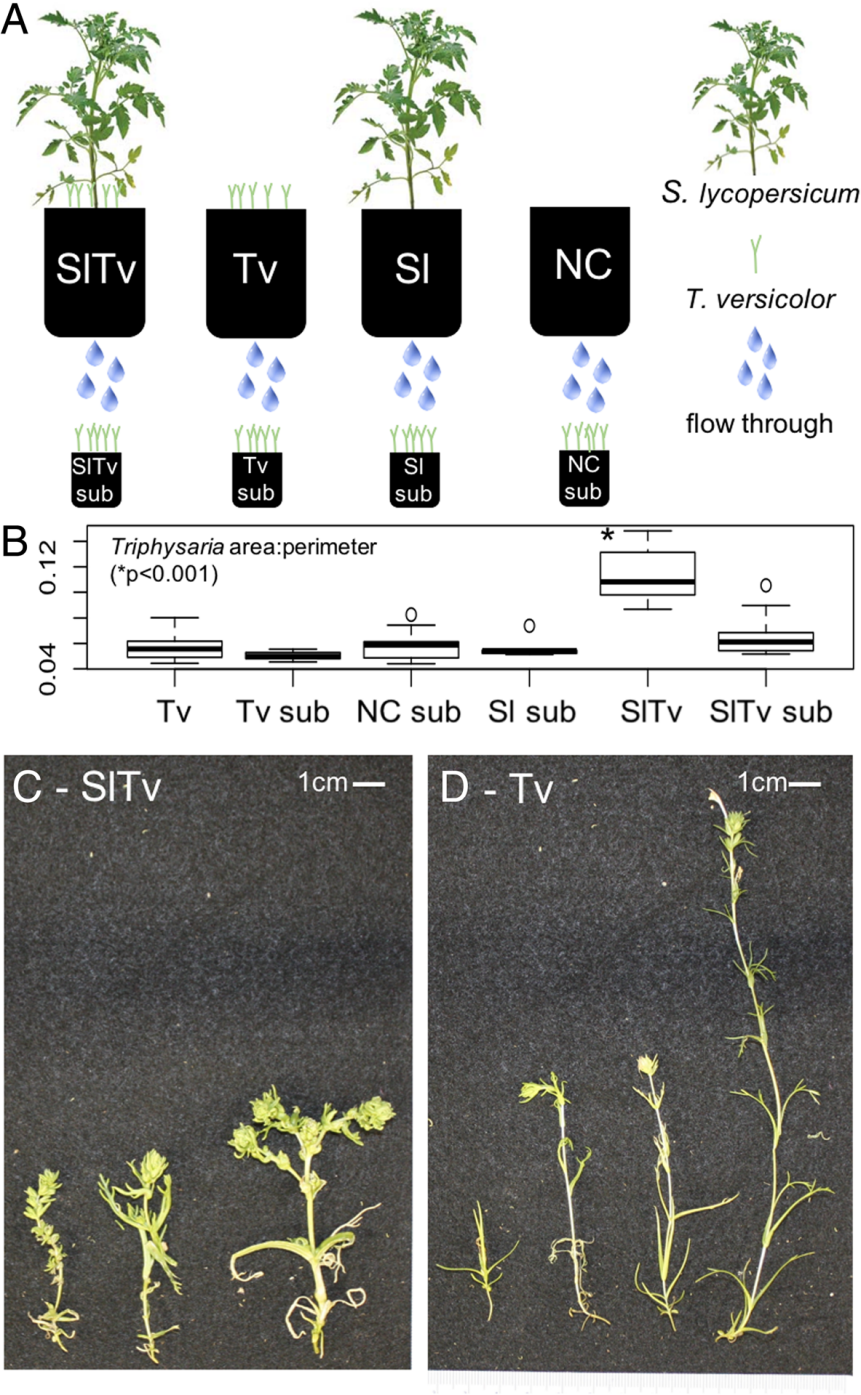
The “plump” phenotype is dependent on direct host contact**. a** experimental design, Sl = *S. lycopersicum,* Tv = *T. versicolor*, NC = negative control; **b** box plot of area:perimeter ratios for all parasites in the experiment; **c & d** example *Triphysaria* images from pot SlTv (parasite + host) and Tv (parasite only) showing the “plump” phenotype that parasites display when grown with *S. lycopersicum* hosts. ANOVA (Tukey-Kramer correction) *p < 0.001

Observations in the first co-culture experiment led to hypotheses about the cause of the host-dependent phenotypes. We sought to separate stimuli that induced the growth phenotypes, so we designed an experiment to isolate signaling cues that involved top watering larger co-culture pots as in the multi host experiment, but instead *collecting* the flow through and then using it to sub-irrigate smaller pots containing *Triphysaria* only (Fig. 3a). We hypothesized that the “pale and plump” phenotype was a result of successful parasite attachment. Therefore, we predicted that this phenotype would be absent from the sub-irrigated pots which lacked direct host contact, yet these plants would be exposed to water soluble host exudates that have been shown to induce haustoria in *Triphysaria* [34]. Indeed the only parasites in the experiment that showed the phenotype were the *Triphysaria* that were grown in direct contact with the tomato host (Fig. 3b SlTv vs. others: see 3c & 3d for representative plant images).

There was a very weak pattern (paired t-test *p* = 0.04) that suggested parasite survivorship was lower in SlTv Sub (27 ± 9% surviving *Triphysaria when* watered with flow through from tomato only pots) than NC Sub (50 ± 6% surviving *Triphysaria when* watered with flow through from soil only pots), yet when we corrected for multiple comparisons the result was not significant. Thus, the survivorship of parasites in this experiment was not significantly different, possibly for one or more of three reasons: 1) growing conditions were cooler, hence more favorable causing more co-cultured parasites to establish, 2) host-free plants, which, although they were more likely to survive without hosts, still showed a trend of decreasing survivorship and thus had more time to die (8 vs. 5 weeks), and 3) lack of statistical power – the control group was roughly 1/5 of the size of the multi-host experiment, which we designed with a very large control group (*n* = 45) based upon a power analysis from preliminary experiments (data not shown). Importantly, parasites in the sub-irrigated pots did not display the plump phenotype, supporting our hypothesis that host contact was required for this distinct phenotype.

### Differentially expressed genes in autotrophic vs. heterotrophic *Triphysaria*

The Parasitic Plant Genome Project (PPGP; [35]; http://ppgp.huck.psu.edu) hosts a publicly available compendium of life stage specific transcriptomes for species including *Phelipanche* (syn. *Orobanche*) *aegyptiaca*, *Striga hermonthica* and *Triphysaria versicolor* (see Yang et al., 2015). The observations we have made with *Triphysaria* feeding across its host range suggest that the parasite’s physiology is substantially altered in a host-dependent fashion. The PPGP transcriptome database includes data for *Triphysaria* grown with and without a host (*M. truncatula*) for flowers/reproductive structures, shoots, and roots. We compared these previously analyzed digital gene expression datasets [13] to find gene activity that differed significantly between the autotrophic vs. heterotrophic modes of the facultative parasite (Table 1).

**Table 1:** GO enrichment of differentially expressed genes in the feeding parasite support the observed host dependent phenotypes**.** Bold numbers indicate *P* < 0.05, adapted from [13]

GOSlim Term	DE genes		*p*-value
Vegetative Shoots	upreg	downreg	
ATPase activity	**28**	4	6.26E-06
peptidase activity	**19**	1	2.22E-05
carbohydrate metabolic process	**13**	3	1.78E-02
response to stress	**13**	2	5.98E-03
nucleus	**12**	2	1.09E-02
translation	3	**15**	2.76E-03
intracellular	3	**16**	1.50E-03
thylakoid	2	**33**	1.24E-09
protein complex	2	**42**	6.07E-13
photosynthesis	1	**28**	6.88E-09
structural constituent of ribosome	1	**15**	1.43E-04
ribosome	1	**15**	1.43E-04
Reproductive Shoots			
peptidase activity	**20**	4	9.56E-04
ion binding	18	**51**	1.21E-06
oxidoreductase activity	6	**41**	3.16E-09
transmembrane transport	3	**14**	5.03E-03
transport	3	**19**	2.22E-04
transmembrane transporter activity	3	**16**	1.50E-03
translation	2	**12**	5.18E-03
response to stress	1	**8**	1.74E-02
structural constituent of ribosome	1	**8**	1.74E-02
ribosome	1	**8**	1.74E-02
Roots			
ion binding	**46**	16	3.37E-05
peptidase activity	**37**	2	2.34E-10
response to stress	**23**	1	1.21E-06
cellular protein modification process	**22**	2	1.70E-05
kinase activity	**20**	2	6.66E-05
biosynthetic process	**19**	8	4.13E-02
DNA metabolic process	**17**	6	2.76E-02
DNA binding	**16**	6	4.35E-02
hydrolase activity, acting on glycosyl bonds	**11**	2	1.95E-02
lipid metabolic process	**9**	1	1.93E-02
translation	1	**11**	2.36E-03
intracellular	1	**10**	4.65E-03

Not surprisingly, genes related to photosynthesis with the GO Biological Process term “photosynthesis” and Cellular Component term “thylakoid” are under-represented in *Triphysaria* when it feeds on *Medicago* compared to the autotrophic (host-free) mode of growth. Consistent with our previous work examining all parasite life stages [13], the Molecular Function GO term “peptidase activity” was overrepresented in the feeding parasite’s root tissue and the Biological Process GO term “translation” was underrepresented among differentially expressed (DE) genes. The GO Biological Process terms “biosynthetic process” and “carbohydrate metabolic process” are notably higher, respectively in root and shoot, in the feeding parasite compared to fully autotrophic *Triphysaria.* Consistent with elevated mortality rates in our experiment that suggest increased parasite stress, both in the roots and shoots of feeding parasites, “response to stress” category genes were strongly upregulated. These gene expression signals are correlated with altered growth patterns and provide candidate genes and processes to examine in future experiments.

## Discussion

### Interpreting the responses of a generalist parasitic plant to a range of hosts

Previous work has suggested that other facultative generalists in Orobanchaceae may show host preference or selectivity [36–38], an observation widely made of parasitic angiosperms [39, 40]. Therefore, in order to gain insight into the host evaluation process, we set out to establish a framework to observe phenotypic clines and transitions associated with host exposure across the parasite’s confirmed host range. Our observations of *Triphysaria* shoots display a spectrum of phenotypic characteristics along the host range of the parasite.

Typically, parasitic plant success is defined as a successful connection to a suitable host [41]. Because we (unpublished field and lab observations) and others [31] have noted transitions in parasite growth patterns that are thought to occur after successful attachment of parasites to host roots (via haustoria), we reasoned that similar obvious transitions in our experiment could be used as a proxy for successful attachment of *Triphysaria* to a suitable host.

*Triphysaria* plants show a range of a “pale and plump” phenotype that is more pronounced on certain hosts than others. This parasite phenotype resulted from shortened internodes and stubbier, fleshier, and more pale leaves. Follow-up analyses of the parasite’s anatomy may provide some additional insight in the processes that drive these growth patterns. Of particular interest would be changes in leaf anatomy, as it is known that the related obligate parasites *Striga gesnerioides* and *Alectra orobanchoides* display diminished leaf morphology compared to free living relatives [42–45]. While the overall height and dry mass of the heterotrophic individuals showed no clear trend, when used to calculate a mass:length ratio, it revealed a clear gradation that might be useful as a proxy for success of the parasite. This is because parasites that displayed the most dramatic phenotypes (grown on *Zea* and *Solanum,* see Fig. 2) accrued more biomass compared to the more gracile individuals grown on other hosts. Indeed, our observations are consistent with previous work in a closely related facultative hemi-parasite [31, 32] as *Triphysaria* also displayed host dependent survivorship as well as host-dependent growth characteristics. Additionally, the distinct paleness of the “plump” individuals is concordant with significant under-representation of genes related to photosynthesis in the feeding parasite, suggesting increased heterotrophy compared to fully autotrophic *Triphysaria*. Together these data show that the hemi-parasite *Triphysaria* displays clear host-dependent phenotypes that are suggestive of variable parasite success, or perhaps even host selectivity, though more work on this question is needed. In fact, Atsatt noted that facultative members of Orobanchaceae (syn. Scrophulariaceae) were ideal candidates to characterize host-specific parasite responses, in part because of the frequently observed enhanced vigor and more rapid growth after a presumed attachment to a host plant [31].

The host dependent phenotypes suggest increased parasite vigor, even though reduced overall survivorship for these heterotrophs was observed compared to more gracile host-free controls in our 5-week co-culture experiment. These observations are consistent with work by Atsatt, who found the closely related facultative parasite, *Castilleja densiflora* (syn. *Orthocarpus densiflora*) has *initially* low survivorship when grown with hosts versus without, but after 2 months the parasites with hosts were more likely to survive [32]. Therefore, the initial low survivorship may reflect a gamble that pays off for successful parasites late in their life cycle when water stress increases late in the season in the northern California part of the parasite’s native range [32]. A larger (necessarily due to high parasite mortality) and longer controlled study, perhaps in the field, would help determine if the same long term trends are seen with *Triphysaria* when feeding on various hosts*.*

The low survivorship of discernably more successful individuals seems to indicate that, like recent work in pea suggests [46], these plants are engaging in risky behavior possibly by allocating resources away from autotrophic modes of growth. This risky behavior may be buffered or canalized by host plants for successfully attached parasites – like the increased survivorship of inbred albino *Orthocarpus purpurascens* (syn. *Castileja* exserta) when grown with a host versus without [47]. It is therefore possible that the parasites die when attempting to transition to heterotrophic modes of growth. In this way *Triphysaria* may be engaging in risk in a similar way as a forager on a negative energy budget [48] – when resources are so limited that survival is unlikely, risk prone behavior in the form of a gamble for a big payoff (in this case a successful union with a host plant root) may be the only viable strategy.

These data shed light on a long-held hypothesis for which relevant data has been very limited – in fact Heide-Jørgensen makes the argument that the distinction between facultative and obligate parasitism is irrelevant because definitive evidence for facultative parasitism does not yet exist and is very hard to obtain [41]. He argues that a fully autotrophic mode of growth in a host-free system may be an artifact of highly favorable growth conditions in the lab that do not reflect growth conditions for facultative parasitic plants in nature. We contend that if autotrophy were a viable strategy, the plants would likely *not* risk costly haustorium formation on hosts that significantly increase mortality. Conversely, we have observed what seem to be autotrophic *Triphysaria* growing and flowering ~ 1 m away from any potential host plant in the field (unpublished). So while these data suggesting risky behavior by *Triphysaria* when grown with known hosts does support the hypothesis that *Triphysaria* is functionally an obligate parasite [41], definitive support for this or the alternative hypothesis remains elusive.

We did attempt to excavate pots from the multi-host experiment to survey haustorium formation, but the very dense, wet, and sandy soil made this extremely difficult. While we frequently observed haustoria, we were unable to attribute connections to certain individuals, or even accurately count the tiny ~ 1 mm haustoria. Development of a co-culture system that would induce the phenotypes we observed and that also allows researchers to monitor haustorium development with ease is needed.

Previous observations have shown that root exudates are sufficient to induce haustorium formation in *Triphysaria* [30]. We therefore attempted to determine if the plump phenotype was due to exudates, induced tomato defenses, or direct host contact. We confirmed that the “plump” phenotype was dependent upon direct contact with a known host, supporting the hypothesis that the phenotype co-occurs with a switch to heterotrophy, not simply exposure to host exudates or allelopathic compounds. Although the signal was very weak that *Solanum* exudate is sufficient to reduce survivorship, this hints that survivorship is linked to haustorium formation and can be induced without direct host contact. Using our image-based analytics, it should be possible to longitudinally monitor plant growth, and select individuals to excavate to search for haustoria when a significant contrast appears in the non-destructive analysis.

## Conclusions

Characterization of the elusive molecular dialogue between parasitic plants and their host plants requires a tractable framework. To that end, we have created a framework that includes non-destructive methods for longitudinal studies and demonstrated that significant differences in easily quantifiable growth patterns are obtainable. Furthermore, these patterns of parasite growth and survival do shed light on long held questions about whether true facultative parasites exist in nature – our data suggest that *Triphysaria* is engaging in risky behavior to potentially parasitize certain hosts more than others. Our framework was optimized with hosts that have resources for molecular genomics work; this will facilitate next steps to explore mechanisms of host choice or evaluation by parasitic plants, as well as the nature of the unique plant-plant molecular dialogue between parasitic Orobanchaceae and their hosts.

## Methods

### Co-culture across *Triphysaria’s* host range

#### Host plants selection

Putative hosts were selected from surveys by Thurman [5] and were further refined to include plants with publicly available genome or transcriptome sequence data resources anticipating molecular studies that leverage this experimental framework. Host plants selected for this project were *Arabidopsis thaliana* (Col-0), *Medicago truncatula* (A17)*, Solanum lycopersicum* (Heinz 57)*, Zea mays* (B73)*, Oryza sativa* subspecies Japonica cv. Nipponbare*,* and *Juncus effuses*. Haustorium formation resulting from host contact was confirmed for each host (Additional file 1: Figure S1).

#### Seed germination

*Triphysaria versicolor, Medicago truncatula* (A17)*,* and *Zea mays* (B73) seeds were obtained and germinated as described by [49]. *Solanum lycopersicum* (Heinz 57) seed were produced in the Penn State Biology Greenhouse. *S. lycopersicum* seeds were surface sterilized using a 50% bleach (5.25% hypochlorite) + 0.01% Triton X-100 (Sigma) solution for 30 min, then washed 10x with sterile distilled water and germinated on *Triphysaria* co-culture medium (1/4x Hoagland’s basal salt and nutrient mix, 7.5 g/L sucrose, 6 g/L plant tissue culture grade agarose, pH of 6.1). *Oryza sativa* subspecies Japonica cv. Nipponbare seeds were incubated in sterile distilled water at 28–37 °C until germination then transferred to *Triphysaria* co-culture medium. *Arabidopsis thaliana* (Col-0) seeds were surface sterilized using 70% ethanol + 0.01% Triton X-100 for 8 min, then washed with 100% ethanol, air-dried for ~ 15 min, and germinated on *Triphysaria* co-culture medium. *Juncus effusus* seed was obtained from a commercial vendor (http://www.everwilde.com/). *J. effusus* seed was washed 2–3 times with sterile distilled water, washed with 70% ethanol for 3–5 min, washed with undiluted commercial bleach (5.25% hypochlorite) for 40 min, washed with sterile molecular biology grade water 3–5 times, and germinated on *Triphysaria* co-culture medium.

#### Experiment layout and watering

The host range co-culture experiment was conducted in the College of Agricultural Sciences Greenhouse #111 at Penn State University from March 3 to May 19, 2014. The experiment was set up in a complete randomized block design (randomizer.org) with 27 pots in each of 5 blocks. Each block contained 6 hosts × 3 replicates plus 9 control [no host] pots each with 7 parasites. A custom drip irrigation system was made using watering timers (Orbit Digital 2-Outlet timer Model #: 27133), ¾” (~ 19 mm) irrigation tubing and various couplers and fittings widely available at hardware stores (Additional file 2: Figure S2). Weighted irrigation drippers were fitted into the ¾” irrigation tubing and standard garden hose ball-check valves were calibrated to flow 100 mL/minute of water to each drip line outlet. Soil media was Sunshine mix #1 and sand (Quickcrete Medium - Lowes) mixed 1:1 (by volume) and measured volumetrically into pots with a triple layer of newspaper in the bottom of each pot to prevent media loss. *Solanum, Maize* and *Oryza* were planted in large pots (2.5 gal, ~ 9.5 L) and received 200 mL of water twice per day, while *Triphysaria* (controls), *Arabidopsis, Medicago* and *Juncus* were planted in small pots (1 gal, ~ 3.8 L) and received 100 mL of water twice per day. The watering regime was calibrated to through-water pots with excess water to maintain high levels of soil moisture. 5 mL granular Osmocote Plus (15–9-12) was added to each irrigated pot and sticky cards were used to control insect pests during the experiment.

#### Planting timeline

After germination (time on plates: *Juncus* 17d, *Medicago* and *Maize* 8d, *Arabidopsis* 7d, *Solanum* and *Oryza -* 6d) host plants were transplanted into soil media (see above) 2 weeks prior to co-culture and grown in a growth chamber (21 °C, 16/8 light/dark cycle) for 1 week (flats with domes for 3 days, with “cracked” domes for 4 days). 1 week prior to transplant to experimental pots, host plants were hardened off in the greenhouse where the experiment took place (3 days with “cracked” domes, 4 days without domes). ~ 6000 *Triphysaria* were germinated and grown in tandem batches at 16 °C ~ 25–35 days prior (*Triphysaria* germination is not highly synchronous) to co-culture. 1 day after host plants were added to experimental pots, germinated *Triphysaria* were transplanted serially (i.e. a researcher transplanted a suitable parasitic plant (defined as ≥1 cm root and first true leaves open) in one pot, then moved to the next pot, until the usable parasitic plants were exhausted). Seven *Triphysaria* plants were added to each host pot ~ 25 cm from the host stem, in a circle, at intervals of roughly 50° (~ 2.5 cm spacing) and *Triphysaria* only pots were planted in the same configuration sans host in small pots (see above). After initial planting the watering regime was supplemented by extensive hand misting with DI water (as needed during the first week ~ 4–5 times daily) to help all plants establish in the greenhouse. Survivorship at week 2 was very high and not significantly different for any treatment, indicating successful establishment.

#### Data collection

The experiment was monitored daily. A survey of survivorship was taken at 1 week intervals. When *Triphysaria* die, they rapidly oxidize and wilt, so our definition of “survivor” was “upright *Triphysaria* with some green leaf tissue”. This definition could include dying individuals, though once senescence had begun, the plant was always counted as dead at the next week interval. On May 16, 2014, 5 weeks after the beginning of co-culture, all parasitic plants were harvested by cutting *Triphysaria* at the soil surface. All plants were photographed (Nikon D80) and then numbered and bagged for drying. Plants were dried for 48 h at 65 °C, at which time each *Triphysaria* was weighed, replaced into the bag, and sealed in an airtight storage container. Plant height was calculated using ImageJ (http://imagej.nih.gov/). Each photograph was analyzed manually by measuring the length, in pixels, of 1 cm on the reference scale bar. These values were averaged, and this value was used to calibrate (86.05 ± 0.12 pixels/cm) the measurement function in ImageJ. The calibrated measurement tool was then used to measure the length from the cut plant (at soil surface) to the apex of each *Triphysaria* plant. To describe the “plump” phenotype observed in the experiment, we normalized the dry mass (in mg) of each *Triphysaria* by the height (in mm).

#### Data analysis

Statistical analysis was done using SAS PROC MIXED [50] as a randomized complete block design with block as a random effect. Dunnett’s test [51] for differences between the treatments (hosts) and the control (no host) was done for each response to control for multiple testing. The unequal allocation of replicates was done to optimize the power of Dunnett’s test. Each of the response variables was analyzed separately.

### Co-culture with *Solanum lycopersicum* and sub-irrigation of *Triphysaria*

#### Seed germination

*Solanum lycopersicum* (Heinz 57) and *Triphysaria versicolor* seeds were obtained and germinated as described above.

#### Experiment layout and watering

A sub-irrigation co-culture experiment was conducted in the College of Agricultural Sciences Greenhouse #85 at Penn State University in from September 16, 2014 to December 14, 2014. Treatments (10 each of *Solanum, Solanum* + *Triphysaria*, *Triphysaria* only*,* soil only control, for a total of 40 pots) were randomly arranged (randomizer.org) and placed in two columns of 20. A drip irrigation system like that described above was used to water each large pot (2.5 gal, 4 L soil media) except that only one timer was used to regulate water to the whole experiment and calibrated to deliver 250 mL of water twice daily to each pot. These irrigated pots were placed at the high end of slightly inclined trays. One small pot (300 ml) with 7 *Triphysaria* only were placed one each at the low end of the inclined trays (that also contained the large pot) so as to be sub-irrigated with the flow-through of the larger pot. A small drainage port in each tray allowed for excess flow-through water to be drained away avoiding water logging of these smaller pots.

#### Planting timeline

*Solanum* and *Triphysaria* were germinated and grown prior to co-culture as described above. Co-culture and control pots were set up as described above. During the first week, watering was calibrated up (from 200 to 250 mL) to deliver sufficient water to sub-irrigated pots. During this optimization phase, dead *Triphysaria* were replaced as needed. Supplemental lighting from overhead sodium vapor lamps was supplied from 8 am to 8 pm if light intensity dropped below 200μE. After the watering regime was established, 5 mL granular Osmocote Plus (15–9-12) was added to each top irrigated pot.

#### Data collection

The experiment was monitored daily and data was collected as described above with the following exception: the duration of the experiment was ~ 8 weeks (59 days) instead of 5 weeks.

#### Data analysis

Statistical analysis was done using SAS PROC MIXED [50] taking into account the pairing of the large and small pots. Tukey’s test for all pairwise comparisons was applied for each response to control for multiple testing.

### Non-destructive, image-based analysis

#### Image acquisition

Images were taken with a D80 Nikon camera on a tripod at a fixed distance from the imaging stage, with fixed focus. Auto exposure was used to compensate for changing light conditions throughout the day in the greenhouse. The plants were processed iteratively through each block. Because each block was randomized the effect of dynamic lighting conditions would affect treatments randomly. The imaging stage had a dark felt background and metric/SAE rulers were fixed to the stage on both X and Y axes.

#### Image segmentation and processing to estimate perimeter:area ratio

All image segmentation was performed using ImageJ (https://imagej.nih.gov/ij/). Prior to segmentation the RGB images were either broken into separate channels or transformed into other color spaces for easier analysis: HSV, HSI, or CMYK. Once a particular channel was chosen the image was thresholded to capture the plant ROIs. The ROI could then be cleaned using an additional thresholding to capture the background within and outside the plant; this created a more clearly defined edge. From the refined ROI, the Particle Analyzer function was used to measure the area and the perimeter of the plant. The raw RGB data from the ROI was also obtained for the purpose of normalization against a white reference. The raw RGB data from the white reference was sampled and normalized using the grey world method [52].

Color normalization was performed using both the white reference (ruler) and plant ROIs by converting raw RGB values to XYZ, where the white reference was applied to normalize ROI values for chromatic adaptation using Bradford matrices [53–55]. Conversion back to RGB color space provides the corrected values needed for estimation of chlorophyll content through Green/Red ratio [33]. For the ImageJ macro, see Additional file 3.

#### Data analysis

Statistical analysis was done using SAS PROC MIXED [50] using the mean values for the surviving plants in each pot. To account for the different numbers of survivors, weighted analysis was done using the number of surviving plants as weights. Block was included as a random effect. Dunnett’s test [51] for multiple comparisons with a control was done to determine differences between the pots with hosts and the control. Unequal allocation of replicates was used to optimize the power of Dunnett’s test.

### Gene expression analysis and function enrichment test of *Triphysaria*

Transcriptome analysis of *Triphysaria versicolor* (functional annotation and identification of differentially expressed (DE) genes in the parasite during free-living versus parasitic modes of growth) was reported previously [13]. The DE genes were subject to a Fisher’s exact test using GO slim terms for upregulated and down-regulated DE genes as the input table (fisher.test function in R), which identified enriched GO slim terms associated with upregulated or downregulated features for shoots, flowers, and roots; alpha was set to 0.05.

## Additional files

Additional file 1: **Figure S1.** All hosts were verified by direct observation of haustorium formation by *Triphysaria****.*** A) *Arabidopsis*, B) *Juncus*, C) *Medicago*, D) *Oryza*, E) *Solanum*, F) *Zea*. (TIFF 4874 kb)

Additional file 2: **Figure S2.** Images of experimental apparati and planting scheme**.** A) Seven *Triphysaria* were planted around each host equidistant from each other and the host plant. For control pots, the arrangement was identical, except without host plants. B) the watering control system. (TIFF 6785 kb)

Additional file 3: Archive containing the ImageJ macro. (IJM 2 kb)

## Data Availability

Transcriptome data is available at http://ppgp.huck.psu.edu.

## Abbreviations

HIF: Haustorium inducing factor
PPGP: Parasitic Plant Genome Project
ROI: Region of interest
