# Supplementary figures and images for: Risk versus reward: host dependent parasite mortality rates and phenotypes in the facultative generalist Triphysaria versicolor
Source: BMC Plant Biol. 2019 Aug 1;19:334. doi: 10.1186/s12870-019-1856-1 (PMC6669981; doi:10.1186/s12870-019-1856-1)

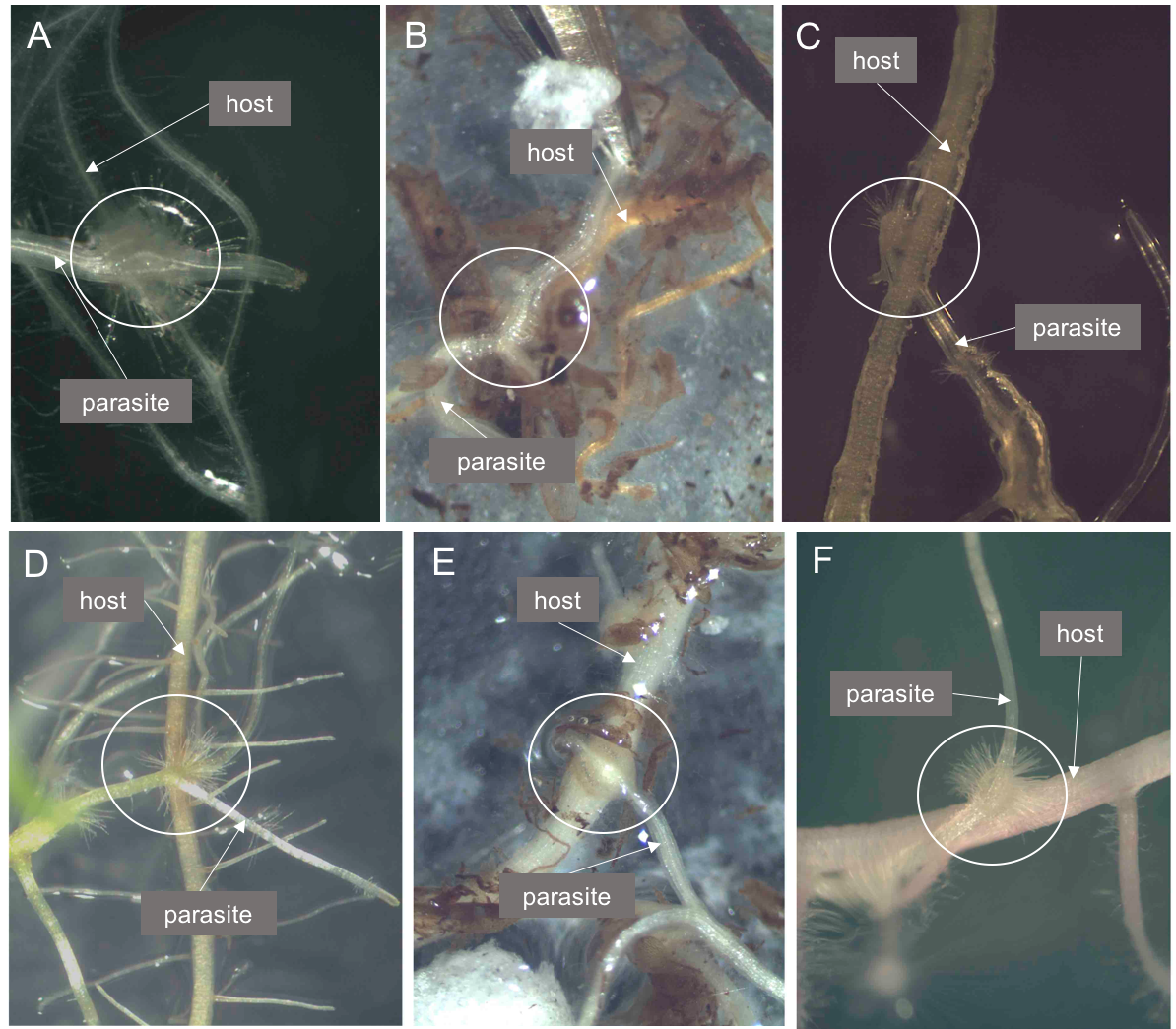

Supplement: Supplementary file 1 — Figure S1. All hosts were verified by direct observation of haustorium formation by Triphysaria. A) Arabidopsis, B) Juncus, C) Medicago, D) Oryza, E) Solanum, F) Zea. (TIFF 4874 kb) [file 12870_2019_1856_MOESM1_ESM.tiff]

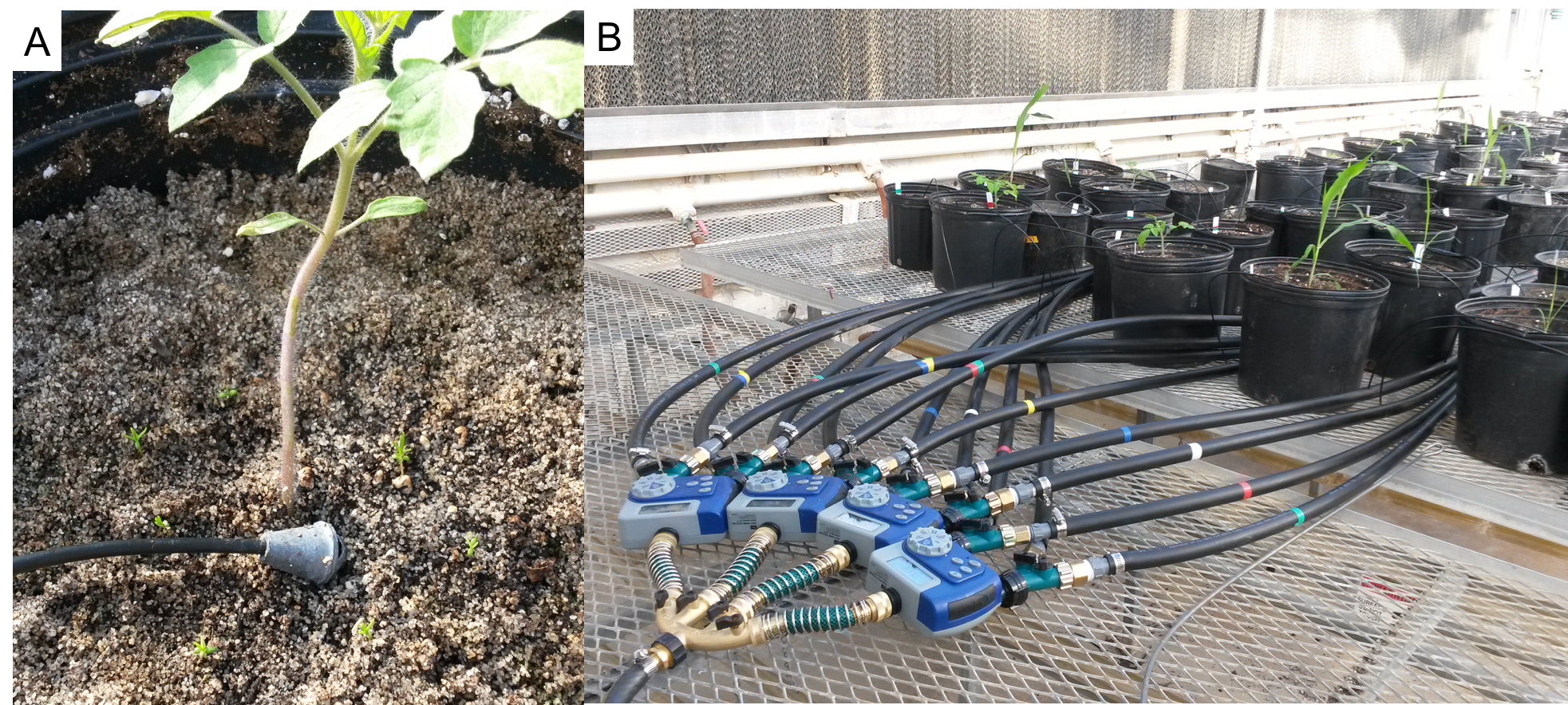

Supplement: Supplementary file 2 — Figure S2. Images of experimental apparati and planting scheme. A) Seven Triphysaria were planted around each host equidistant from each other and the host plant. For control pots, the arrangement was identical, except without host plants. B) the watering control system. (TIFF 6785 kb) [file 12870_2019_1856_MOESM2_ESM.tiff]
